# Biochemical Diversity in the *Trypanosoma congolense* Trans-sialidase Family

**DOI:** 10.1371/journal.pntd.0002549

**Published:** 2013-12-05

**Authors:** Thaddeus T. Gbem, Mario Waespy, Bettina Hesse, Frank Dietz, Joel Smith, Gloria D. Chechet, Jonathan A. Nok, Sørge Kelm

**Affiliations:** 1 Centre for Biomolecular Interactions Bremen, Faculty for Biology and Chemistry, University Bremen, Bremen, Germany; 2 Department of Biochemistry, Ahmadu Bello University, Zaria, Nigeria; Universidade Federal de Minas Gerais, Brazil

## Abstract

Trans-sialidases are key enzymes in the life cycle of African trypanosomes in both, mammalian host and insect vector and have been associated with the disease trypanosomiasis, namely sleeping sickness and nagana. Besides the previously reported TconTS1, we have identified three additional active trans-sialidases, TconTS2, TconTS3 and TconTS4, and three trans-sialidase like genes in *Trypanosoma congolense*. At least TconTS1, TconTS2 and TconTS4 are found in the bloodstream of infected animals. We have characterised the enzymatic properties of recombinant proteins expressed in eukaryotic fibroblasts using fetuin as model blood glycoprotein donor substrate. One of the recombinant trans-sialidases, TconTS2, had the highest specific activity reported thus far with very low sialidase activity. The active trans-sialidases share all the amino acids critical for the catalytic reaction with few variations in the predicted binding site for the leaving or acceptor glycan. However, these differences cannot explain the orders of magnitudes between their transfer activities, which must be due to other unidentified structural features of the proteins or substrates selectivity. Interestingly, the phylogenetic relationships between the lectin domains correlate with their specific trans-sialylation activities. This raises the question whether and how the lectin domains regulate the trans-sialidase reaction. The identification and enzymatic characterisation of the trans-sialidase family in *T. congolense* will contribute significantly towards the understanding of the roles of these enzymes in the pathogenesis of Animal African Trypanosomiasis.

## Author Summary

Trypanosomiasis is a disease also known as sleeping sickness in humans (Human African Trypanosomiasis) and nagana in animals (Animal African Trypanosomiasis). This disease is caused by protozoan parasites of the genus *Trypanosoma*. Tsetse flies are responsible for the transmission of these parasites. *Trypanosoma congolense* is the main causative agent of nagana in cattle. The clinical signs of the disease have been linked to the presence of an enzyme called trans-sialidase. Interestingly, the enzyme alternates in different forms in the mammalian and the insect vector. Previous knowledge had shown that the parasite requires the enzyme for survival in the fly vector. Our current work has revealed other forms of the enzyme that could be essential for the persistence of the disease in mammalian and vector hosts. These enzymes, though similar in structural architecture, show differences in their activities that could be key in delineating their individual roles in the pathophysiology of the disease.

## Introduction

*Trypanosoma congolense* (subgenus: *Nannomonas*) is a major causative agent of the Animal African Trypanosomiasis (AAT) otherwise known as nagana. Other parasites implicated in nagana include *T. vivax* (subgenus: *Duttonella*) and *T. brucei brucei* (subgenus: *Trypanozoon*). These protozoan parasites are transmitted by several species of hematophagous biting flies of the genus *Glossina*. Nagana exhibits a severe negative impact on stock farming, milk and meat production [1]. The impact of the disease is thought to be underestimated as most affected areas are remote, limiting access and hence accurate data acquisition. The need for an alternative arsenal against AAT is heightened as existing drugs are either toxic or rapidly becoming ineffective due to drug resistance [2].

The role of TS in Chaga's disease caused by the South American *T. cruzi* has been extensively studied [3]. On the other hand, studies on the *trans*-sialidases from African trypanosomes responsible for the Human African Trypanosomiasis (HAT) as well as AAT are scanty. In *T. brucei*, TS has been implicated in the cyclical survival of the parasite as evidence supports enhanced survival of parasite in midgut of the insect host [4]. No data exist on *T. congolense* in this regard. Though scanty, the role of blood stream TS and sialidase in anaemia in animals suffering trypanosomiasis caused by *T. congolense* [5] [6] and *T. vivax* [7] has been established.

Multiple copies of TS-like genes exist in *Trypanosoma* genomes. The highest number occurs in *T. cruzi*, but most of the over 1000 genes encod enzymatically inactive proteins [8]. In African trypanosomes, the TS-like gene families are much smaller. For example, 9 members have been identified in *T. brucei* [9] [10] [11]. In *T. congolense*, at least 17 TS-like genes have been identified [12] [13] [14] [6]. Eleven of these, forming the TconTS1 family, are closely related and share over 95% sequence identity [14]. The key element mediating the functions of TS has been ascribed to the N-terminal catalytic domain (CD) harbouring the active site with characteristic conserved amino acids [15] [16] [17] [9] [18], whereas hardly anything is known about possible functions of the lectin-like domain (LD) at the C-terminus of these enzymes.

Here we report that besides TconTS1, three additional members of the *T. congolense* TS gene family transfer sialic acids between glycoconjugates, but have much lower sialidase activities. The identification and biochemical characterisation of *T. congolense* TS genes will enable new studies investigating the role of these genes in nagana disease.

## Methods

Unless where stated, all chemicals and reagents used were cell culture and analytical grade. *Vibrio cholerae* sialidase was purchased from Roche Diagnostics (Mannheim, Germany). *Pfu* DNA polymerase, *Hind*III, *Xba*I, *Spe*I, *Eco*RI and *Dpn*I, PageBlue, molecular weight marker (PageRuler), BCA assay protein kit were all purchased from Thermo Scientific (St. Leon-Rot, Germany). VivaSpin 6 and VivaCell250 ultracentrifugation units were from Sartorius (Göttingen, Germany). Anti-SNAP-tag rabbit polyclonal antibody was from GeneScript (Piscataway, USA) while anti-*Strep*-tag rabbit polyclonal antibody, *Strep*-Tactin resin beads and buffers were from IBA (Göttingen, Germany). Hygromycin and Gentamycin were purchased from PAA, (Pasching, Austria). Polyethylenimin transfection reagent, glucuronic acid, *N*-acetyl-neuraminic acid (Neu5Ac), 3'sialyllactose (3'SL) and lactose were purchased from Sigma-Aldrich (Steinheim, Germany). Ex-cell CD CHO media from SAFC, USA, X-ray film, Enhanced Chemiluminescence system, and recProtein-A Sepharose Fast Flow were purchased from GE Healthcare (Uppsala, Sweden). Polyvinylidene difluoride membrane was from Millipore (Schwabach, Germany).

### Cloning, sequencing, expression and purification of trans-sialidase genes

The Basic Local Alignment Search Tool (BLAST) was used to search the shot-gun sequences of *T. congolense* at the WSTI (http://www.sanger.ac.uk). Using the BLASTN algorithm, the “*T. congolense* reads” were queried with the partial nucleotide sequences (Genbank Accession numbers TS1: AJ535487 and TS2: AJ535488) previously described [13]. Perfect BLAST hits (smallest sum probability P(N)<10-10) were arranged into contiguous sequences using Contig Express (Invitrogen, Carlsbad, USA). By searching the database with ends of the contiguous sequences, the assembled contigs were expanded until open reading frames (ORF) were obtained. On the basis of the obtained ORFs, primers (Supporting Information, Table S1) were designed to amplify by nested PCR the ORF including flanking regions encoding for TconTS2, TconTS3 and TconTS4 using genomic DNA of *T. congolense* strain STIB249 [13]. The resulting products were cloned into the pBlueScript KS- vector (Stratagene, Santa Clara, Ca, USA) via *Spe*I and *Bam*HI (TconTS2) or via *Eco*RI and *Sma*I (TconTS4) or into the mammalian expression vector pcDNAIII Amp (Invitrogen, Carlsbad, USA) via *Hind*III and *Xba*I (TconTS3) and sequenced (Supporting Information, Table S2).

Cloning and sequencing of *T. brucei* TS genes followed a similar strategy as described for *T. congolense* above except that genes were cloned in pJET1.2/blunt vector (Thermo Scientific) following instructions of the manufacturer (for primers see Supporting Information, Table S1).

For the expression of secreted TconTS proteins in mammalian fibroblasts, corresponding DNA sequences without those encoding the signal peptides and GPI anchors were subcloned into a modified pDEF vector providing a 3C protease recognition site, SNAP and *Strep* tags using *Spe*I and *Bam*HI restriction sites [14]. For this purpose, the *Bam*HI site in TconTS3 as well as the *Spe*I and *Bam*HI sites in TconTS4 were removed by site directed mutagenesis without changing the amino acid sequence encoded (for primers see Supporting Information, Table S1). All sequences and mutations were confirmed by Sanger dideoxy DNA sequencing at the Max Planck Institute for Marine Microbiology, Bremen, Germany.

Recombinant TconTS proteins were purified as described [14]. Briefly, CHO_Lec1_ cells (ATCC CRL-1735) were transfected with polyethylenimine, transfection reagent (Sigma, Steinheim, Germany) and stably expressing cell lines selected with hygromycin. Expression of recombinant protein was tested from cell culture supernatant by SDS-PAGE and Western blots methods using rabbit anti-*Strep* and anti-SNAP antibodies. CHO_Lec1_ cells producing TconTS proteins were subsequently adapted to chemically defined Excel CD CHO media.

### Purification of anti-TS1 monoclonal antibody

The 7/23 hybridoma cells [12] were grown for 3 days in RPMI media supplemented with IgG depleted 10% FCS. The tissue culture supernatant was cleared by ultracentrifugation at 105×g for 60 min and anti-TconTS antibody was purified using recProtein-A Sepharose Fast Flow and eluted with 0.1 M glycine/HCl pH 3.0. Antibody containing fractions were neutralised with 1M Tris pH 8.0 and dialysed against 10 mM phosphate buffer. Purified antibodies were used in the detection of TconTS proteins in SDS-PAGE and Western Blot analysis as described [14].

### Trans-sialidase and sialidase reactions

Purified recombinant proteins were assayed for sialidase and TS activities using Neu5Ac-MU and fetuin as sialic acid donor substrates and lactose as acceptor substrate as described before [14]. In brief, reactions of 50 µL containing substrates and enzymes were incubated at 37°C for the times indicated. Sialidase activity was determined as free sialic acids released from Neu5Ac-MU, 3'SL or fetuin in the absence and/or presence of an acceptor substrate. TS activity on the other hand was determined as 3'SL produced in the presence of lactose. Both, free Neu5Ac and 3'SL were quantified using high performance anion exchange chromatography with pulsed amperometric detection (HPAEC-PAD) using the Dionex system, DX600 (Dionex Sunnyvale, CA, USA) [14]. The curve fit module of SigmapPlot 11 was used to calculate v_max_ and K_M_ employing the Michaelis-Menten equation v = v_max_×C_s_/(C_s_+K_M_).

### Phylogenetic analysis

For the phylogenetic analysis TconTS1b, TconTS2, TconTS3 and TconTS4 were aligned with TS and sialidase sequences from *T. brucei*, *T. vivax*, *T. cruzi* and *T. rangeli*. As outgroup the sialidase from *Vibrio cholerae* was used (genes listed in Supporting Information Table S2). Full length protein sequences were first aligned using ClustalW in Geneious and then truncated at the N-terminus. CDs started from the FRIP region to the N-terminus of the conserved α-helix (HL) linking the CD to the LD. The LDs were taken immediately after the α-helix linkage to the C-terminus without the stop codon. DNA sequences encoding either full length proteins, the CDs or the LDs were aligned based on the alignment obtained for the amino acid sequences by T-Coffee algorithm in RevTrans, version 2.0 (http://www.cbs.dtu.dk/services/RevTrans-2.0/web/). DNA sequences used in the phylogenetic calculations are shown in Supporting Information Files S1, S2 and S3. Best parameters (HKY substitution model with 6 gamma rate categories) for phylogenetic constructions were determined using MEGA5 and applied in the phylogenetic calculations using the “MrBayes” plug-in of Geneious.

## Results

### *T. congolense* sialidase/*trans*-sialidase genes

Partial coding sequences of TconTS1 and TconTS2 genes had been described [13]. From “reads” of the WTSI *T. congolens*e genome sequencing project (http://www.sanger.ac.uk), we assembled the full length sequences coding TconTS1 and TconTS2. Further BLAST hits with smallest sum probabilities (P(N)<1–10) were identified and arranged into contiguous sequences leading to further five genes with sequence similarities. Two of the putative gene products shared over 40% sequence identity with TconTS1 and TconTS2 and contained all the conserved amino acids required for transfer reactions [17] [18] [10]. Consistent with the naming of TconTS1 and TconTS2 [13], we refer to them as TconTS3 and TconTS4. The other three genes were distantly related with 20–30% amino acid identity (Table 1) and lack several of the conserved amino acid residues. We assume that these set of genes are likely without sialidase or TS activity and were named TconTS-Like1, TconTS-Like2, and TconTS-Like3.

**Table 1:** *Trypanosoma congolense trans*-sialidase sequence similarities expressed as percentage of identical amino acids in pair-wise alignments.

*Trans*-sialidase	TconTS2	TconTS3	TconTS4	TconTS-Like1	TconTS-Like2	TconTS-Like3
TconTS1	42.2%	43.6%	46.2%	21.1%	26.3%	29.8%
TconTS2	-	48.3%	42.8%	20.8%	26.2%	29.3%
TconTS3		-	48.9%	21.1%	25.1%	29.9%
TconTS4			-	21.3%	27.8%	29.8%

Tcon = *Trypanosoma congolense*

**Figure 1:**
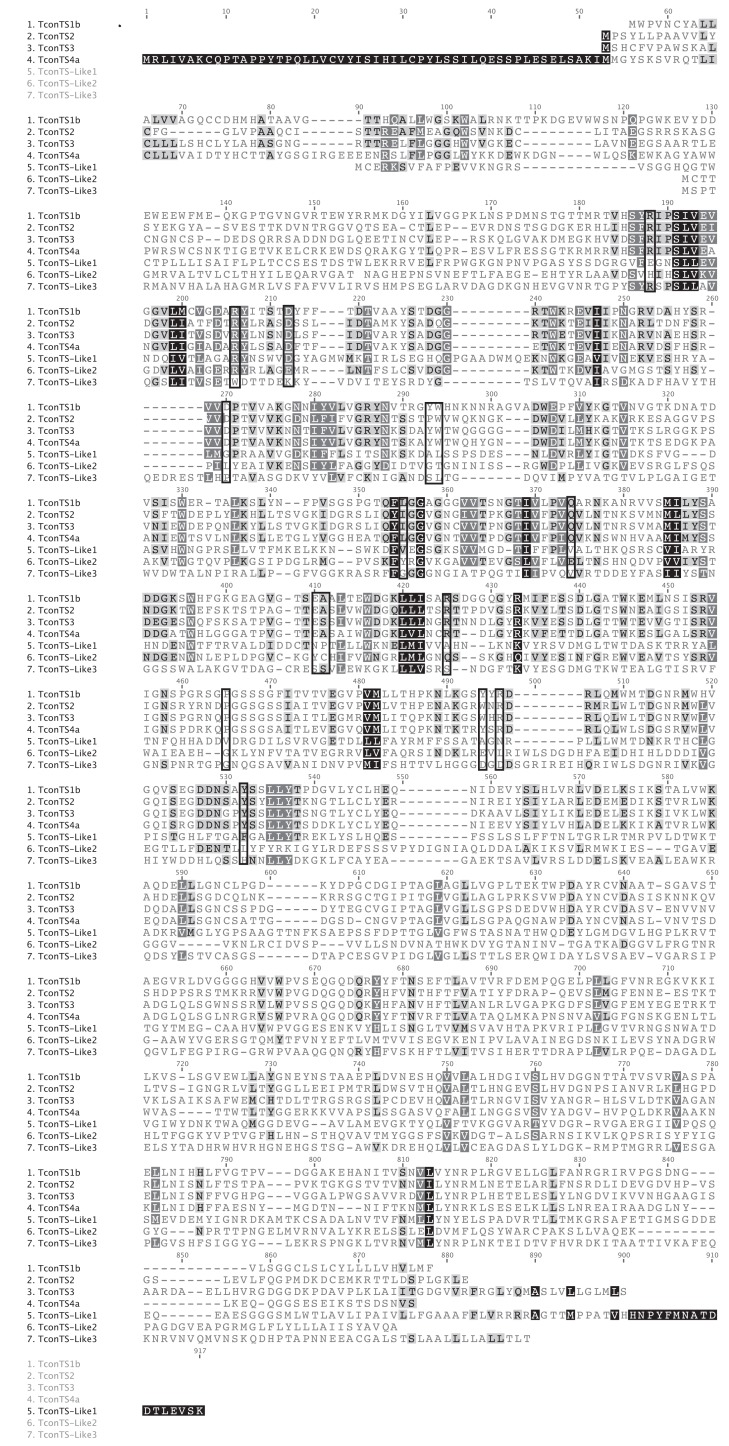
Primary amino acid sequence alignment of TconTS and TconTS-Like genes. Amino acid sequences of TconTS1b (EMBL:HE583284), TconTS2, TconTS3 and TconTS4 were obtained by sequencing of cloned genes. Those of TconTS-Like 1, TconTS-Like 2 and TconTS-Like 3 were obtained from the WSTI database. CluwstalW alignment plugin of the Geneious software was used employing BLOSUM12 with gap openings and extension penalties of 10 and 0.1, respectively. Increasing darkness of background indicates increasing numbers of identical amino acids at each position. The numbers on top of the sequences indicate the positions in the consensus sequence. Amino acid residues postulated to be critical for catalysis, substrate binding and structure as given in Table 2 are boxed.

In order to compare sequence similarities between TconTS genes, we cloned and sequenced full lengths TconTS2, TconTS3 and TconTS4. In an earlier study, we amplified eleven highly similar (about 96% identical amino acids) but clearly different sequences of TconTS1 from *T. congolense* genomic DNA [14]. Sequencing several clones of TconTS2, TconTS3 and TconTS4 provided no evidence for such heterogeneity of these genes. The alignment of these genes is given in Figure 1.

Amaya *et al.* [18] identified amino acids in *T. cruzi* TS involved in the catalytic and substrate binding. Whereas these amino acids are not conserved in the three TconTS-like gene products, they are almost completely conserved in TconTS1, TconTS2, TconTS3 and TconTS4 (Table 2). Most of these are conservative changes, with the exception of position 293 (numbering in the consensus sequence), where a Tyr is replaced by Pro in TconTS2. Furthermore, based on mutagenesis experiments [19] [17] with *T. cruzi* TS, two proline residues corresponding to positions 411 and 465 appear to be required for full TS activity. Whereas at position 465 Pro is conserved across the TconTS, it is not found in the TconTS-like gene products (Table 2). However, at position 411 the Pro is not conserved in TconTS, but replaced by Ala or Ser.

**Table 2:** Amino acids in the catalytic domains of TS and TS-Like genes from *T. congolense* involved in enzymatic activities*.

Consensus	Tcon TS1	Tcon TS2	Tcon TS3	Tcon TS4	Tcon TS-Like1	Tcon TS-Like2	Tcon TS-Like3
catalysis							
212	D150	D135	D142	D207	D110	E85	K86
410	E324	E309	E316	E381	N291	Y257	S262
532	Y438	Y423	Y430	Y493	F404	L375	H382
substrate binding							
188	R126	R111	R118	R183	E86	H61	R62
425	R339	R324	R331	R396	A306	Q272	S277
496	R410	R395	R402	R465	N375	L339	D346
206	R144	R129	R136	R201	R104	R79	W80
270	D188	D173	D180	D245	G160	L122	P132
293	Y211	P196	Y203	Y268	A183	G145	S155
294	W212	W197	W204	W269	L184	T146	L156
374	Q289	Q274	Q281	Q364	V255	E222	V227
494	Y408	W393	W400	Y463	A373	E337	D344
structure							
411	A325	A310	S317	A382	P292	C258	S263
465	P379	P364	P371	P434	V344	G308	G315

* The indicated amino acids have been selected based on structural [18] and mutation [19] [23] studies with *T. cruzi* TS and on the sequence alignment of TconTS1b with *T. cruzi* [14]. Amino acid positions have been numbered based on the consensus of alignment (Figure 1) or starting methionine of each ORF.

### TS orthologues occur in *T. congolense* and *T. brucei* but not in *T. vivax*

**Figure 2:**
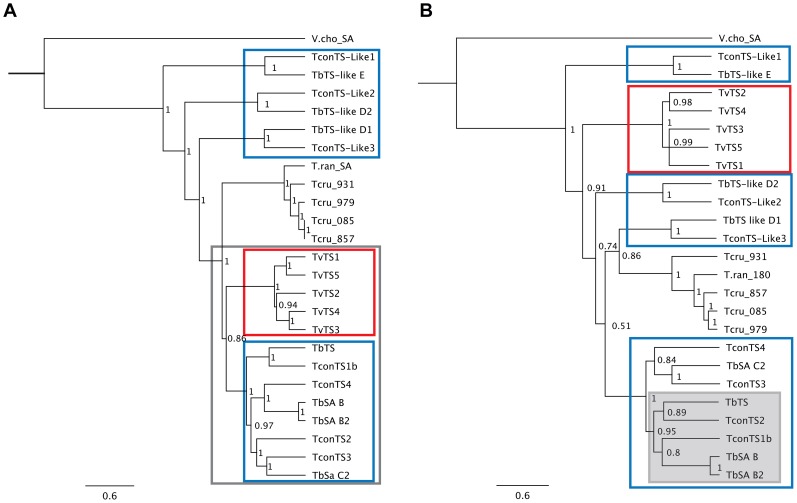
Phylogenetic analysis TS genes. Phylogenetic analyses of DNA sequences were performed as described under Methods using “MrBayes” plug-in of Geneious. Trees are midpoint rooted and nodes supported by posterior probability values and non-parametric bootstraps generated by maximum likelihood analysis in “MrBayes” as described under Methods. TS genes from *T. congolense* and *T. brucei* are marked by blue frames, TS genes from *T. vivax* by red frames. **A**: Phylogenetic tree for full ORFs, the “African TS-branch” is marked by a grey frame; **B**: Phylogenetic tree for LDs, the most active TconTS and their orthologues from *T. brucei* are highlighted by a grey box.

To decipher the phylogenetic relationship between TS and TS-like genes of African trypanosomes, we compared the four TconTS and three TconTS-Like sequences together with seven sequences from *T. brucei* and five from *T. vivax* (Supporting Information Table S2) using the alignment of DNA sequences reverse transcribed from the protein alignment (see Supporting Information Files S1, S2 and S3 for DNA sequences used). As shown in Figure 2A, for each TconTS and TconTS-Like gene a corresponding orthologue was identified in *T. brucei*, whereas *T. vivax* gene products cluster separately from TS of the other African trypanosomes. TconTS-Like2 and TconTS-Like3 form a branch together with their *T. brucei* orthologues separate from all South American TS genes. In contrast, TconTS-Like1 and its *T. brucei* orthologue appear to be more closely related with the more distant South American branch than the African genes.

Trypanosomal TS contain an N-terminal CD followed by a C-terminal LD. Besides the phylogenetic analysis with the entire ORFs, analysis were performed using sequences coding for each domain separately. The phylogenetic tree obtained for the CDs resembles that for complete ORFs (not shown). Surprisingly, a different situation was observed for the LDs of TconTS1, TconTS2, TconTS3 and TconTS4 and the *T. brucei* TS genes (Figure 2B). First, within the TconTS genes, the LD of TconTS2 is most closely related to that of TconTS1, whereas the CD of TconTS3 is more closely related to TconTS2. Second, it should be noted that not the same *T. congolense* and *T. brucei* genes group as orthologous pairs, if LDs are compared. Amplification and sequencing ORF of *T. brucei* TS genes confirmed that the combination of the CDs and LDs were as predicted from the contigs in the databases.

### Monclonal anti-TS1 antibody cross-reacts with TconTS2, recognising an epitope on the lectin domain

**Figure 3:**
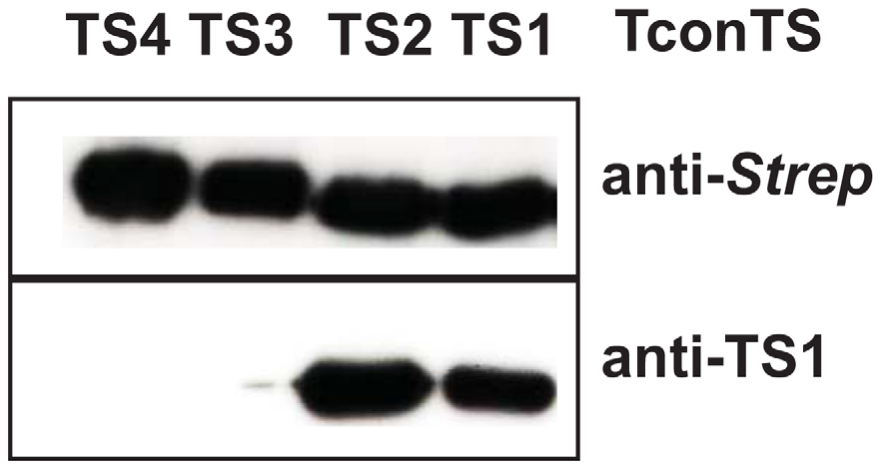
Recognition of TconTS proteins by antibodies. 100 ng of affinity purified TconTS proteins were analysed by Western blot as described under Methods. Blots were probed either with polyclonal rabbit anti-Strep (upper pane) or with monoclonal mouse anti-TS1 antibody, which shows cross reactivity with TconTS2 (lower pane).

To biochemically characterise TconTS genes, recombinant proteins were made for TconTS2, TconTS3 and TconTS4 as previously described for TconTS1 [14]. Recombinant TconTS proteins were expressed in CHO_Lec1_ [20] and purified by affinity chromatography to obtain pure protein from eukaryotic cells with high mannose-type *N*-glycans. The apparent molecular masses of the recombinant TconTS proteins including the SNAP and *Strep* tags are between 110 and 125 kDA as resolved on SDS-PAGE. The generic *Strep* tag fused to the proteins is recognised by anti-*Strep* polyclonal Ab in all the recombinant TconTS proteins as shown in Figure 3 (upper panel). Surprisingly, the monoclonal anti-TS antibody [12] reacted with both TconTS1 and TconTS2, but not TconTS3 and TconTS4 (Figure 3; lower panel). This result points to a similar epitope being present in both TconTS1 and TconTS2. Further experiments provided evidence that the epitope is located in the LDs.

### TconTS2, TconTS3 and TconTS4 are trans-sialidases

**Figure 4:**
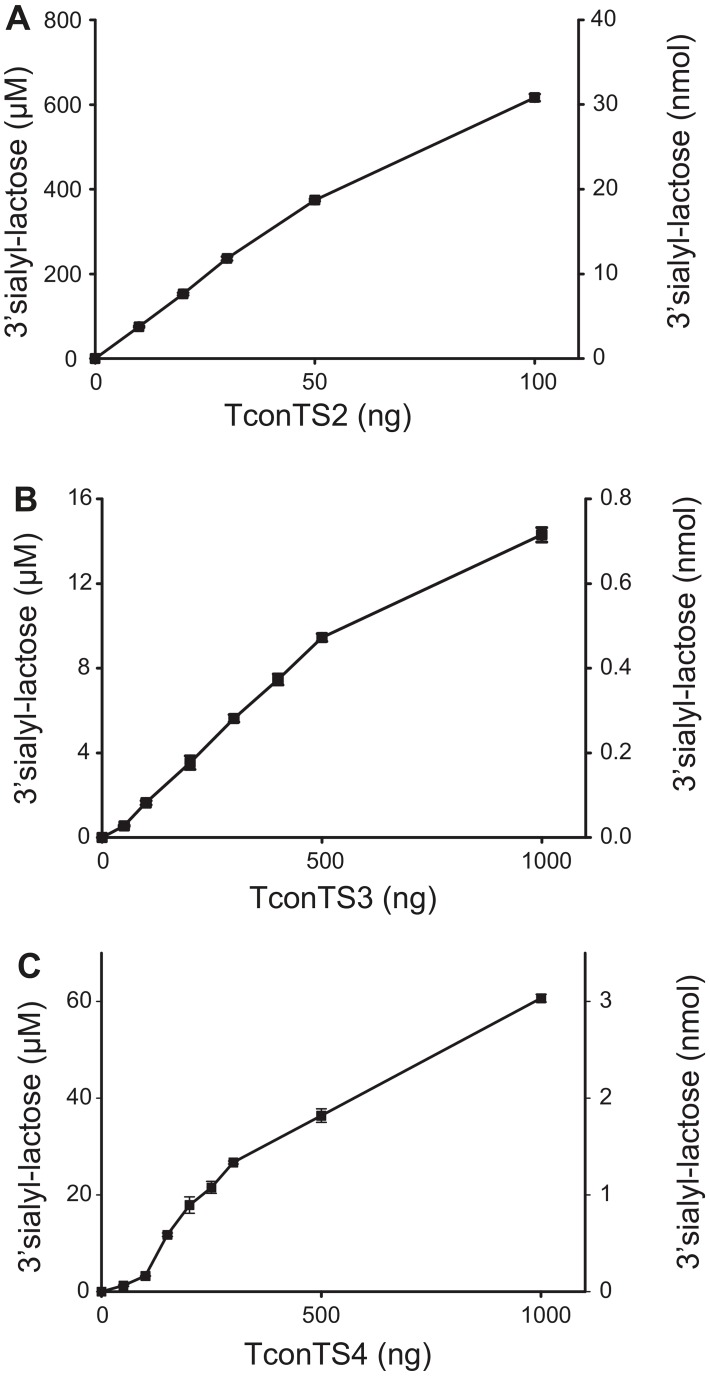
Concentration dependency of TS activity. The indicated amounts of recombinant TconTS proteins were incubated with 100 µg fetuin (600 µM bound Sia) and 2 mM lactose for 30 (TconTS2), 240 (TconTS3), or 1440 (TconTS4) minutes. 3'SL produced was determined by HPAEC-PAD as described under Methods. Data points are means ± standard deviations of three independent experiments, each as triplicates. **A**: TconTS2, **B**: TconTS3, **C**: TconTS4.

In order to investigate the enzymatic activities we used the glycoprotein fetuin as donor and lactose as acceptor substrates. Free sialic acid (the product of sialidase activity) and 3'SL (the product of TS activity) could be quantified simultaneously from HPAEC-PAD chromatograms of the reactions. Under standard conditions 25 ng TconTS2 produced about 330 pmol/min 3'SL leading to 200 µM 3'SL in the reaction mix within 30 minutes (Figure 4A). Product formation by TconTS2 was linear for up to 50 ng enzyme under these conditions. The reaction catalysed by TconTS3 was slower than that of TconTS2, since 500 ng of enzyme generated only 4.2 pmol/min 3'SL corresponding to 10 µM 3'SL after 4 h (Figure 4B). 3'SL formation by TconTS3 was almost linear for 4 h. TS activity was also detected for TconTS4. However, the activity was even lower than that of TconTS3 and 500 ng of TconTS4 produced less than 0.1 pmol/min 3'SL (Figure 4C). Therefore, 24 h incubations were routinely used to determine TconTS4 activity.

To determine kinetic parameters (Table 3) reactions were conducted under standard conditions for 30, 120 and 1440 minutes for TconTS2, TconTS3 and TconTS4, respectively. TconTS2 exhibited the lowest K_M_ for fetuin (299 µM), similar to that reported for TconTS1b (395 µM) [14], whereas those determined for Tcon TS3 (6090 µM) and TconTS4 (949 µM) were higher. The lowest K_M_ for lactose was found for TconTS2 (602 µM), followed by TconTS3 (1104 µM) and TS4 (1806 µM). Comparing the results with those obtained for two variants of TconTS1 [14], TconTS2 has a K_M_ value for lactose similar to TconTS1e-1 but about 3-fold less than TconTS1b. For both substrates, TconTS2 showed about 2-, 200- and 2000-fold higher v_max_ values than TconTS1, TconTS3 and TconTS4, respectively.

**Table 3:** Kinetic parameters of TconTS.

	Donor substrate (fetuin-bound Sia)*		Acceptor (lactose)**	
	v_max_ *** (µmol/(min×mg TS))	K_M_ *** (µM)	v_max_ *** (µmol/(min×mg TS))	K_M_ *** (µM)
TS1b****	7.9±0.3	359±45	4.3±0.1	1683±101
TS1e-1****	7.6±0.5	1617±223	2.1±0.1	727±48
TconTS2	17.62±0.13	299.00±7.0	17.85±0.13	602±16
TconTS3	0.17±0.02	6090.00±1267	0.0567±0.0014	1104±79
TconTS4	0.0067±0.0002	949±50	0.0075±0.0002	1806±112

* Approximately 30 nmol Sia per 100 µg fetuin; 2 mM lactose was used as acceptor substrate.

** 600 µM fetuin-bound Sia was used as donor substrate.

*** K_M_ and v_max_ were calculated from Michaelis-Menten kinetics (see Supplementary Information, Figure S1) by SigmaPlot. Data points are mean ± standard deviations of three independent experiments, each replicated thrice.

**** values from Koliwer-Brandl et al. [14].

### Sialidase activities

**Figure 5:**
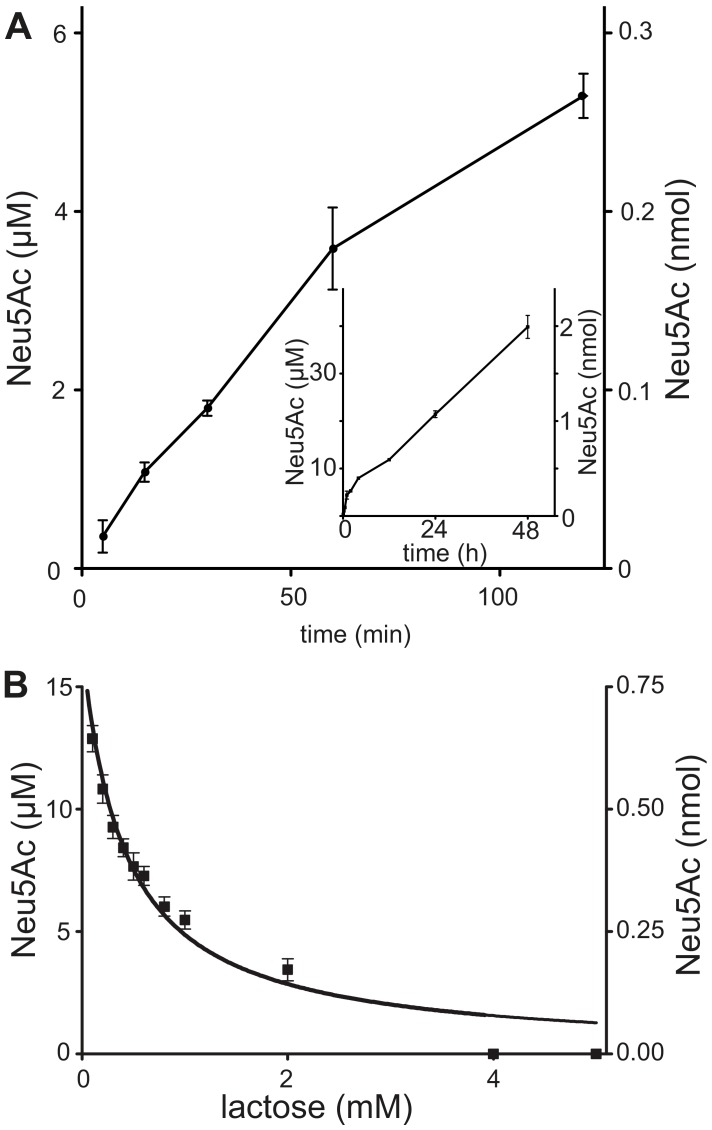
Sialidase activity of TconTS4. **A**: Recombinant TconTS4 was incubated with 100 µg fetuin (600 µM bound Sia) for the times indicated (see insert for long term reactions) and free Sia determined by HPAEC-PAD as described under Methods. Data points are means ± standard deviations of 3 independent reactions each triplicated. **B**: Recombinant TconTS4 was incubated for 1440 min with 100 µg fetuin (600 µM bound Sia) in the presence of the lactose concentrations indicated and free Sia determined by HPAEC-PAD as described under Methods. Data points are means ± standard deviations of 3 independent reactions each as triplicates.

Sialidase activity has been shown for TconTS purified from *T.congolense* axenic culture [12] and for *T. congolense* infected animals [5] [7]. Therefore, we investigated the sialidase activities of TconTS using fetuin as a model glycoprotein. Whereas no release of free Neu5Ac was observed for TconTS1, TconTS2 and TconTS3 under standard conditions of TS assays, TconTS4 clearly showed sialidase activity producing 0.76 pmol/min Neu5Ac up to 48 h (Figure 5A). Indeed, the sialidase activity of TconTS4 is relatively stable and retained a residual sialidase activity of 40% even after incubation at 37oC for 120 days.

We also investigated the effect of lactose on TconTS4 sialidase activity. At lactose concentrations above 3 mM, release of Neu5Ac dropped to undetectable levels (Figure 5B). This result indicates the existence of a competition between lactose and water for the cleaved Neu5Ac from the donor fetuin. This is confirmed by the increased amount of 3'SL produced with increasing lactose concentration.

When we incubated TconTS2 or TconTS3 with fetuin and lactose for extended periods, it was observed that increasing amounts of Neu5Ac were released, similar to TconTS1 [14]. Interestingly, free Neu5Ac appeared only after 3'SL has accumulated as a product of transialylation. For example, in TconTS2 reactions, Neu5Ac was detectable (0.68 µM, 27 pmol) when the 3'SL concentration had reached almost 600 µM, the maximum 3'SL concentration reached. Whereas further incubation did not result in higher 3'SL concentrations, the amount of free Neu5Ac continuously increased. This observation suggests that TconTS2releases free Neu5Ac from 3'SL but not from fetuin. Similar observations were made for TconTS1, but the highest 3'SL concentration reached was about 300 µM. These data indicated that for TconTS1 and TconTS2 at 300 µM and 600 µM 3'SL, respectively, the transfer of Neu5Ac between fetuin and 3'SL has reached an equilibrium. For TconTS3, we could not reach such equilibrium; probably since the maximum 3'SL concentration obtained was 50 µM due to the low specific activity of this enzyme. Nevertheless, small amounts of free Neu5Ac were detected in prolonged TconTS3 reactions leading to 50 µM 3'SL. Also in this case, Sia appears to be released from 3'SL only, since in the absence of lactose no release of free Neu5Ac could be observed.

These observations suggest that TconTS1, TconTS2 and TconTS3 can release free Neu5Ac from glycoconjugates, but the level of product released is too low to be detected under standard TS assay conditions. To address this, higher amounts (500 ng) of TconTS1, TconTS2, TconTS3 and TconTS4 were incubated with 600 µM fetuin-bound Neu5Ac without lactose for 2 h. Under these conditions sialidase activities could also be detected for TconTS1 and TconTS2, but not for TconTS3 (Table 4). However, compared to the corresponding TS activities, the sialidase activities were very low.

**Table 4:** Sialidase activities of TconTS.

	Sialidase* (nmol/(min×mg TS))	Trans-sialidase** (nmol/(min×mg TS))	TS/sialidase
TconTS1	59.7±2.3	4,000±100	67
TconTS2	73.5±4.0	17,850±130	243
TconTS3	n.d.***	34±1	>30
TconTS4	2.7±0.07	9±0.2	3.4

* Sialidase activities were determined by incubating 500 ng of the TconTS indicated for 2 h with 100 µg fetuin (600 µM fetuin-bound Sia). Data points are mean ± standard deviations of three replicates.

** TS activity values see Table 3.

*** n.d. not detected.

## Discussion

The enzymatic properties of four TconTS were compared using fetuin as a model for a blood glycoprotein donor substrate. Two of these enzymes, TconTS1 and TconTS2, exhibit about 100- or 1000-fold higher specific TS activities than TconTS3 and TconTS4, respectively. The K_M_ values for lactose were around 1 mM (0.6 to 1.8 mM) for all four TconTS and did not correlate with their specific activities. The K_M_ values for the donor fetuin were more different ranging from 0.4 to 6 mM glycoprotein bound sialic acids. The K_M_ for fetuin also did not correlate with the specific activity, since the highest K_M_ was determined for TconTS3, one of the enzymes with low activity, and TconTS4 has a similar K_M_ as TconTS1, but is 1000-fold less active. Since the K_M_ values are lower than the substrate concentrations used, especially for TconTS4, the specific activities given in Table 3 are lower than those to be expected, if the acceptor substrate lactose would be at saturating concentrations.

Tiralongo *et al.* [12] purified two TconTS forms with different TS/sialidase activity ratios from procyclic *T. congolense* cultures. Peptide sequences from these preparations have demonstrated that they contained at least TconTS1 [12] [14]. Recombinant TconTS1 variants expressed in eukaryotic cells had lower specific activities for synthetic substrates than described for the purified enzyme [12] suggesting that these TconTS preparations also contained other enzymes and/or factors influencing the TS/sialidase activities [14]. For example, in those preparations, Glutamic Acid-Rich Protein, GARP, a natural substrate for TconTS was co-purified with one of the TS forms [12]. Although it is unclear what role GARP might have played, its presence, as well as other TS enzymes, might have been responsible for the reported higher specific activities of these TS preparations for synthetic substrates. In summary, it appears that TconTS1 and TconTS2 are responsible for most of the TS activity of *T. congolense*. However, it may well be that TconTS3 and TconTS4 are more active on other donor substrates, such as glycoproteins and/or glycolipids from blood components, the natural substrates for trypanosomal TS.

It has been established that procyclic forms of African trypanosomes express TS [21] and emerging evidences point to expression also in the blood stream forms [6] [7]. So far, no information is available on which TS genes are expressed at what stage of the parasite's life cycle. Recently, we have identified mRNAs for TconTS1, TconTS2 and TconTS4 in the blood of infected goats (data not shown). Also the stability and persistence of shed enzymes in the blood stream has to be taken into account. *In vitro* TconTS1 and TconTS3 are the most stable of the four enzymes investigated, retaining full TS activities even after four months at 37°C. Under these conditions TconTS2 lost its activity completely, while TconTS4 retained 40% residual sialidase but no transfer activity (data not shown). It would be interesting to investigate whether this long-term stability correlates with a sustained persistence of enzyme activity in the blood stream.

Lactose was found to suppress the sialidase activity of TconTS4. *In vitro*, lactose appears to be a better acceptor than water (Figure 5B). Therefore, in presence of lactose, the transfer activity of TconTS4 is more efficient than its hydrolytic activity. Anaemia in animals suffering African *Trypanosoma* infections has been attributed to the effects of sialidases [22] [5] [7]. Desialylation of erythrocytes by sialidases exposes underlying galactose residues and their subsequent degradation. The presence of lactose in mammalian blood would lead to lowered efficiency of parasites sialylation and eventual clearance by the immune system. However, desialylation of parasites is equally possible in presence of lactose due to the action of TS. Along this line it is interesting to note that infusion of lactose in the blood of sheep suffering experimental anaemia from *T. congolense* suppressed anaemia (unpublished observation).

All amino acid residues shown to be involved in the catalytic reaction or interaction with the substrate for *T. cruzi* TS are conserved in the TconTS enzymes (Table 2). Only the two residues interacting with the methylumbelliferyl aglycon or the lactose part of 3'SL in the *T. cruzi* TS [18], positions 293 and 494 in consensus sequence (Figure 1), are different in the two most active TconTS1 and TconTS2. This could explain why these enzymes do not use Neu5Ac-MU as substrate (data not shown). Furthermore, these changes could lead to a weaker interaction with the leaving groups and thus facilitate their release during catalysis. In this context it is interesting to note that the most drastic change, Tyr to Pro at position 293, occurs as P196 in TconTS2, the most active enzyme with the highest TS/sialidase ratio (Table 4). Certainly, this modification will reduce the interaction with hydrophobic aglycons or the leaving galactose residue of the donor substrate.

Amaya *et al.* [18] also provided evidence that in *T. cruzi* TS Y119 (position 293 in the consensus sequence) also contributes to hydrogen bonding with O9 of the covalently bound Sia following a conformational change induced by the reaction. Such an interaction would not be possible in TconTS2, but could be compensated by hydrogen bonding with the conserved side chains W197 and Q274 of TconTS2 corresponding to W120 and Q195 in *T. cruzi* TS, two amino acids contributing to the hydrogen bonding network of O9 in the covalently bound Sia [18].

Similarly, although to a lesser degree, the replacement of a Trp at position 494 (corresponding to W312 in *T. cruzi* TS) with a Tyr, as found in TconTS1, is expected to reduce the hydrophobicity of this site leading to a reduced affinity for the leaving group. In *T. cruzi* TS substitution of this Trp (W312) by Ala basically abolished Neu5Ac transfer but only slightly decreased hydrolytic activity for 3'SL [23]. Interestingly, in contrast to the wild type *T. cruzi* TS, this mutant was not able to hydrolyse Neu5Ac-MU, similar to TconTS1, which also does not accept Neu5Ac-MU as a substrate [14].

It appears that TS activity depends on well controlled conformational changes [17] influenced by specific proline residues. This is supported by the potential of the *T. rangeli* sialidase to acquire transfer ability due to a change of Gln to Pro at position 284 [24] and the loss of enzymatic activity in *T. cruzi* TS by the reverse mutation [16]. At the corresponding position 465 Pro is found in all active TconTS (Table 2). The relevance of conformational changes in the enzyme rather than a direct specific interaction of the amino acid were also indicated by mutation of Pro231 to Ala in *T. cruzi* TS [19], corresponding to position 411 in the consensus sequence. Although this mutation led to a significant decrease in *T. cruzi* TS activity, all active TconTS have an Ala or Ser at this position. It would be interesting to see, if higher TS activities can be obtained by introducing a Pro at this position, particularly in TconTS3 or TconTS4, the two enzymes with low TS activities.

Besides these critical amino acids listed in Table 2, other structural features obviously control the ratio of TS versus sialidase activities, since TconTS2 and TconTS4 share identical amino acids at all these positions. Yet, TconTS4 has the highest sialidase to TS ratio amongst the TconTS enzymes, whereas TconTS2 has the lowest ratio (Table 4). Koliwer-Brandl *et al.* [14] observed for TconTS1 that a natural mutation that replaced R144 (206 in consensus) sequence with Cys in the variants TconTS1g (EMBL: HE582290) did not terminate but only reduced TS activity, while increasing relative hydrolytic activity. It can be assumed that a weaker interaction with the hydroxyl group at C4 of sialic acid is responsible for the catalytic properties of TS1g, since the Arg (R53 in *T. cruzi* TS) is in close contact with the bound sialic acid and probably supports the stabilisation of the enzyme-substrate complex [18].

Conservation and/or substitution of amino acids in the active centre of the catalytic domain did not give clear indications of activity differences between the TconTS enzymes. In this context, an interesting aspect is how the different specific activities of TconTS enzymes correlate with those of related gene products from other African trypanosomes. Phylogenetic analyses have allowed the clear assignment of orthologues for *T. brucei*, but not for *T. vivax*, where TS genes clustered exclusively together and away from the TS genes of *T. congolense* and *T. brucei* (Figure 2) [7] [25]. A direct comparison of the TS activities between *T. congolense* and *T. brucei* is difficult, since limited comparable data for enzymatic activities is available. In *T. brucei* TbTS and TbSA C2 have been identified as active TS [9] [10] [11]. This is consistent with the observation that their orthologues (TconTS1 and TconTS2, respectively) are the most active TS in *T. congolense*. RNA*i* based experiments provided evidence that in *T. brucei* TS and sialidase activities are encoded by different genes, TbTS and TbSA C [10], whereas assays with purified recombinant proteins demonstrated both enzyme activities for TbTS and TbSA C2 [11]. Noticeably, the diversity of TS-related genes in *T. vivax* is lower than what was obtained for *T. congolense* and *T. brucei*. Equally, *T. vivax* is distinctively different from *T. congolense* and *T. brucei* in terms of development in the insect host. While the later two develop in the insect midgut and proboscis or salivary glands respectively, *T. vivax* develops exclusively in mouthparts of the tsetse. Moloo and Gray [26] showed that *T. vivax* ingested with blood meal to the midgut is disintegrated. TS-like genes from *T. vivax* share all but two (consensus sequence positions 411 and 494) of the conserved amino acids listed in Table 2 with the active TconTS. Recently Guegan *et al.* [7] reported that TvivTS2 has TS activity. Furthermore, they obtained evidence for the presence of at least TvivTS1, TvivTS3 and TvivTS5 in the bloodstream form and none in epimastigotes and the possible involvement of these proteins in anaemia in infected mice. In summary, it may be possible that *T. vivax* is missing a suitable TS to survive and colonise the fly vector midgut. To this end, it would be interesting to see the survival ability of transgenic *T. vivax* expressing a TS, which is expressed by *T. congolense* in the midgut of tsetse flies.

The presence of multiple highly similar TS genes, as described for TconTS1 [14], suggests that these genes undergo active rearrangements, which could lead to strain specific differences. For example, Coustou *et al.* [6] referred to two highly related TconTS3 genes in the IL3000 strain identified in GeneDB and TrytrypDB databases. However, we could not find evidence for their existence in the STIB294 strain used in this study. Similarly, closely related genes with over 80% sequence identity have been identified for TbSA B and TbSA C in *T. brucei* [11].

TS and sialidase genes of African trypanosomes are organised in two major domains; the CD and the LD. The LDs of TconTS are more varied (40% pairwise identity) when compared with the CDs (58% pairwise identity). Surprisingly, the phylogenetic relationships between the TS are clearly different, if only the LDs are included in the analysis (Figure 2). Furthermore, the LDs of the two most active enzymes TconTS1 and TconTS2 are more closely related than the CDs, where TconTS2 is most closely related to TconTS3. Interestingly, the monoclonal anti-TS1 antibody also binds TconTS2, recognising an epitope in the LD. First preliminary experiments obtained with recombinant proteins, in which the LDs have been swapped between TconTS, provided supporting evidence that the LD influences TS and sialidase activities of the enzymes (data not shown). However, the specific activities of these proteins expressed in bacteria is much lower than those of the proteins expressed in fibroblasts described here, suggesting that for conclusive interpretation the domain swapped TconTS have to be expressed in eukaryotic cells and that further studies are necessary to optimise the fusion of the two domains. In summary, these data indicate a more significant role for the LD for the TS activities of TS1 and TS2 and thus possibly in the pathogenesis of African trypanosomiasis.

## Supporting Information

Figure S1 **Trans-sialidase reaction velocities depending on substrate concentrations.** Product (3′-sialyl-lactose) amounts were determined as described under Methods. v_max_ and K_M_ for lactose shown in Table 3 were calculated from these data. Data points are mean ± standard deviations of three independent experiments, each replicated thrice. **A) TconTS2 with different donor substrate concentrations.** 50 ng TconTS2 were incubated for 30 minutes with 2 mM lactose and the indicated concentrations of fetuin-bound Sia. **B) TconTS2 with different acceptor substrate concentrations.** 50 ng TconTS2 were incubated for 30 minutes with 600 µM fetuin-bound Sia and the indicated lactose concentrations. **C) TconTS3 with different donor substrate concentrations.** 250 ng TconTS3 were incubated for 120 minutes with 2 mM lactose and the indicated concentrations of fetuin-bound Sia. **D) TconTS3 with different acceptor substrate concentrations.** 500 ng TconTS3 were incubated for 120 minutes with 600 µM fetuin-bound Sia and the indicated lactose concentrations. **E) TconTS4 with different donor substrate concentrations.** 500 ng TconTS4 were incubated for 1440 minutes with 2 mM lactose and the indicated concentrations of fetuin-bound Sia. **F) TconTS4 with different acceptor substrate concentrations.** 500 ng TconTS4 were incubated for 1440 minutes with 600 µM fetuin-bound Sia and the indicated lactose concentrations.
(PDF)

File S1 **Nucleotide sequences of the sialidase and trans-sialidase genes used in the phylogenetic comparison of “full length open reading frames” (catalytic plus lectin domains).** The gaps inserted for the alignment as described under Methods are indicated by dashes. The file is a text file in FASTA format with the gene names (see Table S2) in the first line for each gene.
(TXT)

File S2 **Nucleotide sequences of the sialidase and trans-sialidase genes used in the phylogenetic comparison of “catalytic domains”.** The gaps inserted for the alignment as described under Methods are indicated by dashes. The file is a text file in FASTA format with the gene names (see Table S2) in the first line for each gene.
(TXT)

File S3 **Nucleotide sequences of the sialidase and trans-sialidase genes used in the phylogenetic comparison of “lectin domains”.** The gaps inserted for the alignment as described under Methods are indicated by dashes. The file is a text file in FASTA format with the gene names (see Table S2) in the first line for each gene.
(TXT)

Table S1 **List of primers used for cloning and mutagenesis.** Listed are the primers used in this study for cloning, expression plasmids and mutagenesis as described under Methods.
(PDF)

Table S2 **Trans-sialidase and sialidase genes used for phylogenetic analysis.** Listed are accession numbers and literature references for the genes used in the phylogenetic analysis in this study.
(PDF)
