# Supplementary figures and images for: Biochemical Diversity in the Trypanosoma congolense Trans-sialidase Family
Source: PLoS Negl Trop Dis. 2013 Dec 5;7(12):e2549. doi: 10.1371/journal.pntd.0002549 (PMC3855035; doi:10.1371/journal.pntd.0002549)

Figure S1

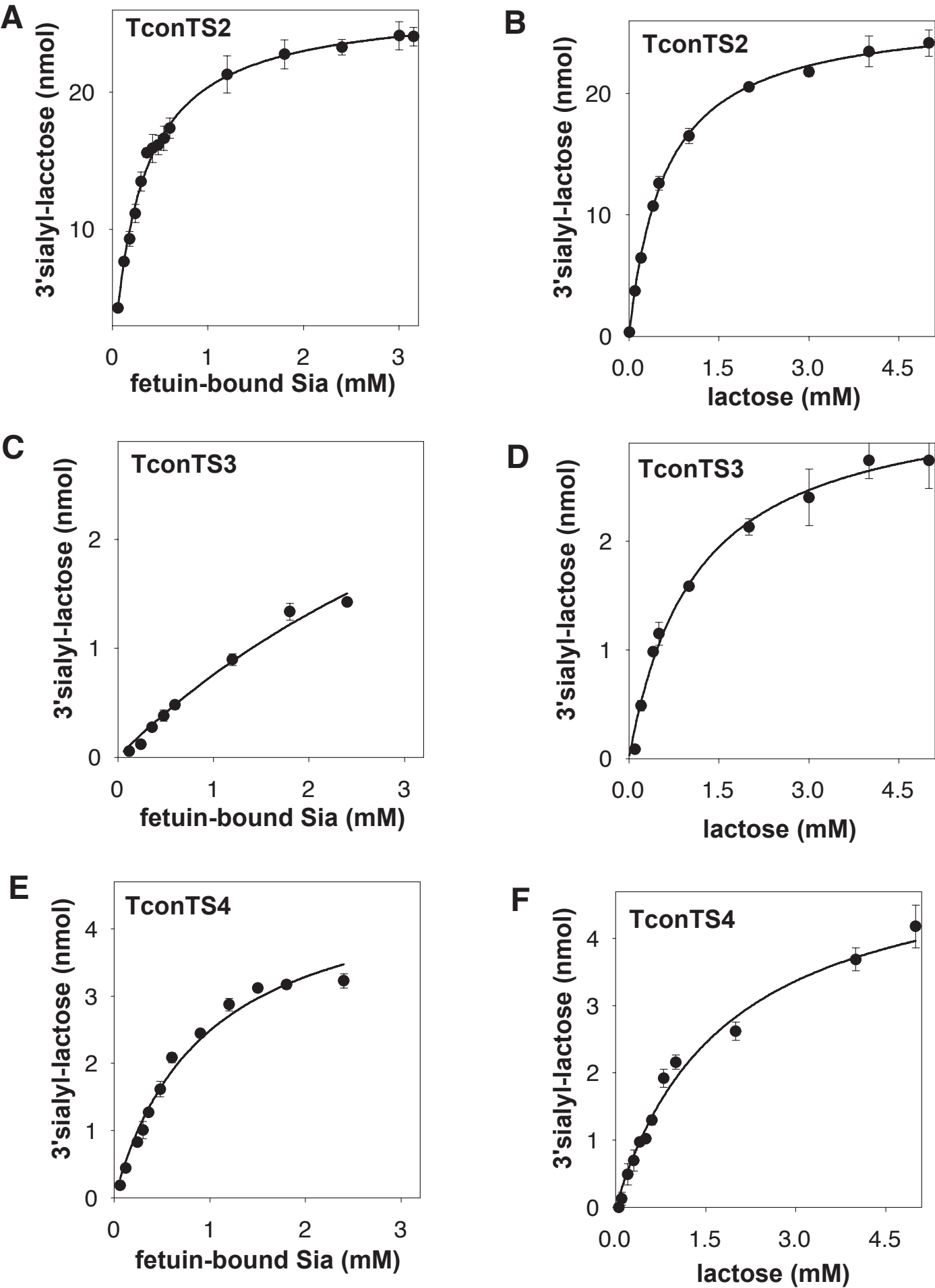

Supplement: Figure S1 — Trans-sialidase reaction velocities depending on substrate concentrations. Product (3′-sialyl-lactose) amounts were determined as described under Methods. vmax and KM for lactose shown in Table 3 were calculated from these data. Data points are mean ± standard deviations of three independent experiments, each replicated thrice. A) TconTS2 with different donor substrate concentrations. 50 ng TconTS2 were incubated for 30 minutes with 2 mM lactose and the indicated concentrations of fetuin-bound Sia. B) TconTS2 with different acceptor substrate concentrations. 50 ng TconTS2 were incubated for 30 minutes with 600 µM fetuin-bound Sia and the indicated lactose concentrations. C) TconTS3 with different donor substrate concentrations. 250 ng TconTS3 were incubated for 120 minutes with 2 mM lactose and the indicated concentrations of fetuin-bound Sia. D) TconTS3 with different acceptor substrate concentrations. 500 ng TconTS3 were incubated for 120 minutes with 600 µM fetuin-bound Sia and the indicated lactose concentrations. E) TconTS4 with different donor substrate concentrations. 500 ng TconTS4 were incubated for 1440 minutes with 2 mM lactose and the indicated concentrations of fetuin-bound Sia. F) TconTS4 with different acceptor substrate concentrations. 500 ng TconTS4 were incubated for 1440 minutes with 600 µM fetuin-bound Sia and the indicated lactose concentrations. (PDF) [file pntd.0002549.s001.pdf]
