# Supplementary material for: Biochemical Diversity in the Trypanosoma congolense Trans-sialidase Family
Source: PLoS Negl Trop Dis. 2013 Dec 5;7(12):e2549. doi: 10.1371/journal.pntd.0002549 (PMC3855035; doi:10.1371/journal.pntd.0002549)
Supplement: Table S1 — List of primers used for cloning and mutagenesis. Listed are the primers used in this study for cloning, expression plasmids and mutagenesis as described under Methods. (PDF) [file pntd.0002549.s005.pdf]

Table S1: List of primers used for cloning and mutagenesis

Gene	Outer forward	Outer reverse	Inner forward	Inner reverse
TconTS2	GTAGAGTTGTCGC GCACCTCG	CATTAACGGACAC AAAGTTA	CGCAAGCTTGACTGA AAGGAAGTCAGTA	CGTCTAGATTAATGTAGCCA TATTATT
TconTS3	AATGGCCCCCTCC CAGAAACAACTC	CACTAATGCTCACA GCCTCCAGT	CGCAAGCTTACTAAGA CGCAAGTTCAAT	CGTCTAGATAATAACACTAC ATTCGCC
TconTS4	AACATTCCACTACA TGTAGTGATA	TTTCGGTTGAACAT TAATTT	CGGAATTCATCCTACA AGAAAGCTC	CGCCCGGGAGTGCGATTG CCATTCCTA
TbTS	ATCCGGACATATCA GC	GGGACAAGAAAAA ACAC	GGAGGAATGGAGGAA C	TACGTACTGGAGAACGTC
TbSA B	CATTGTGAGGAGT GTATAGG	GATGGCACGCATA AC	CGGAGAATATATGAAG C	CAGATTATAGTAGAATCC
TbSA B2	GTGTATTCGCTAG GTGC	GAATGAGCCTGCA CAC	CAGAAAGTATATGGAA CGC	GCATCGGAAATCAAA
TbSA C2	GTTGGTAACCACG GACT	CATTACTCTACGAC AATTAGTG	TTGATCAATGGCATCC	GCTGACAGTAACTGGAGC
TbTS-like D1	GGAAGTATTATTCA TCCCAT	ATTCGTCACACTG CAC	TCAGCTCATTTCGATG	CCGTGTTTTACATAAATAGG
TbTS-like D2	GACTATTTCCGTAC CCTAA	GATTTCTATTTGCA TTCTG	TTCGCAATGAGAATGC	CTGCAGTTTTCAAAAAGTG
TbTS-like E	ATAACCTGTGCATT TGC	CGTATTATCCTCCT CACG	GTACCAGCATGAGAGT ATG	CATGGATTATAGACCTCATT
	**Forward**		**Reverse**	
TconTS1 CD	GCAAGCTTCAGTGCTGCGACCACATG		CGGGATCCATCACGATAATACGAGCCCT	
TconTS1 LD	CGGGATCCACTCGAGCTGCCAGGACCA		CGGGATCCGTCACTCGATTCGAATAT	

	**Mutagenesis**	**Forward**	**Reverse**
TconTS3	Remove *BamH*I site	CACGAGGGGATCTAGGGGCTCTCCT	AGGAGAGCCCCTAGATCCCCTCGTG
TconTS4	*Remove Spe*I site	GCTGCTGTACACCAGTGACGATAAG	CTTATCGTCACTGGTGTACAGCAGC
	*Remove BamH*I site	CTCCCGCGTGGTTGATCCCACAGTT	CTCCCGCGTGGTTGATCCCACAGTT

* Nested PCR amplification with primers in 5' -3' direction, CD = catalytic domain, LD = lectin domain
