# Supplementary material for: Biochemical Diversity in the Trypanosoma congolense Trans-sialidase Family
Source: PLoS Negl Trop Dis. 2013 Dec 5;7(12):e2549. doi: 10.1371/journal.pntd.0002549 (PMC3855035; doi:10.1371/journal.pntd.0002549)
Supplement: Table S2 — Trans-sialidase and sialidase genes used for phylogenetic analysis. Listed are accession numbers and literature references for the genes used in the phylogenetic analysis in this study. (PDF) [file pntd.0002549.s006.pdf]

Table S2: *Trans*-sialidase and sialidase genes used for phylogenetic analysis

Organism	Names*	accession number	Reference
V. cholerae	V. cho_SA	M83562.1	[1]
T. vivax	TvTS1	TvY486_0806830	[2]
	TvTS2	TvY486_0039990	[2]
	TvTS3	TvY486_0024490	[2]
	TvTS4	TvY486_0027090	[2]
	TvTS5	TvY486_0038450	[2]
T. brucei	TbTS	Tb927.7.6850	[3]
	TbSA B	Tb927.5.640	[4]
	TbSA B2	Tb927.7.7480	[5]
	TbSA C2	Tb927.8.7350	[5]
	TbTS-like D1	Tb927.2.5280	[4]
	TbTS-like D2	Tb11.01.3240	[4]
	TbTS-like E	Tb927.5.440	[4]
T. congolense	TconTS1b	HE583284	[6]
	TconTS2	HG531955	this study
	TconTS3	HG531956	this study
	TconTS4	HG531957	this study
	TconTS-Like1	TcIL3000.0.25120	TriTrypDB**
	TconTS-Like2	TcIL3000.0.21990	TriTrypDB**
	TconTS-Like3	TcIL3000.2.1400	TriTrypDB**
T. cruzi	Tcru_931	TcCLB.505931.30	[7]
	Tcru_979	TcCLB507979.30	GeneDB***
	Tcru_857	TcCLB508857.30	GeneDB***
	Tcru_085	TcCLB507085.30	GeneDB***
T. rangeli	Tran_SA	U83180	[8]

names used in Figure 3

** database (http://tritrypdb.org); *** database (http://www.genedb.org)

1. Vimr ER, Lawrisuk L, Galen J, Kaper JB (1988) Cloning and expression of the *Vibrio cholerae* neuraminidase gene *nanH* in *Escherichia coli*. J Bacteriol 170: 1495–1504.
2. Guegan F, Plazolles N, Baltz T, Coustou V (2013) Erythrophagocytosis of desialylated red blood cells is responsible for anaemia during *T. vivax* infection. Cell Microbiol. doi:10.1111/cmi.12123.
3. Montagna G, Cremona ML, Paris G, Amaya MF, Buschiazzo A, et al. (2002) The trans-sialidase from the african trypanosome *Trypanosoma brucei*. European Journal of Biochemistry 269: 2941–2950. doi:10.1046/j.1432-1033.2002.02968.x.
4. Montagna GN, Donelson JE, Frasch ACC (2006) Procyclic *Trypanosoma brucei* expresses separate sialidase and trans-sialidase enzymes on its surface membrane. J Biol Chem 281: 33949–33958. doi:10.1074/jbc.M604951200.
5. Nakatani F, Morita YS, Ashida H, Nagamune K, Maeda Y, et al. (2011) Identification of a second catalytically active trans-sialidase in *Trypanosoma brucei*. Biochemical and Biophysical Research Communications 415: 421–425. doi:10.1016/j.bbrc.2011.10.085.
6. Koliwer-Brandl H, Gbem TT, Waespy M, Reichert O, Mandel P, et al. (2011) Biochemical characterization of trans-sialidase TS1 variants from *Trypanosoma congolense*. BMC Biochemistry 12: 39. doi:10.1186/1471-2091-12-39.
7. Jackson AP, Allison HC, Barry JD, Field MC, Hertz-Fowler C, et al. (2013) A Cell-surface Phylome for African Trypanosomes. PLoS Negl Trop Dis 7: e2121. doi:10.1371/journal.pntd.0002121.s003.
8. Buschiazzo A, Campetella O, Frasch AC (1997) *Trypanosoma rangeli* sialidase: cloning, expression and similarity to *T. cruzi* trans-sialidase. Glycobiology 7: 1167–1173.
